# The Attachment Process of the Mothers of Children with Autism Spectrum Disorders in the Pre-School Years: A Mixed Methods Study

**DOI:** 10.3390/children12091169

**Published:** 2025-09-02

**Authors:** Miran Jung, Kuem Sun Han

**Affiliations:** 1 Department of Nursing, Baekseok University, Cheonan 31065, Republic of Korea; rcuty@bu.ac.kr; 2 College of Nursing, Korea University, Seoul 02841, Republic of Korea

**Keywords:** autism spectrum disorder, child, preschool, attachment behavior, mothers, parent–child relations

## Abstract

Background/Objectives: Autism spectrum disorder (ASD) is characterized by qualitative difficulties in interaction and communication, as well as hyper- or hypo-responsivity to sensory input, which can substantially challenge the formation of mother-child attachment. This study aimed to identify attachment levels among mothers of preschool-aged children with ASD and to delineate the attachment processes associated with those levels, with the goal of developing a grounded theory explaining these processes. Methods: A two-step study using methodological triangulation was conducted. In the first quantitative study, the attachment level of 64 mothers of children with ASD, under the age of 7 years in Korea, were measured. And 12 were selected for a second study using the grounded theory method of Strauss & Corbin. Results: A significant attachment difference (*t* = 4.39, *p* < 0.001) was found in the pregnancy plan. The core attachment category in mothers of pre-school children with ASD was identified as “keep on going with closing the distance”. Eight stages and four types were found in their attachment process. Conclusions: The results of this suggest that it is necessary to develop a personalized intervention strategy and to provide proper nursing by considering the attachment process and type of mothers of children with ASD.

## 1. Introduction

Attachment is a strong emotional bond formed between a child and a parent by its persistent affectionate and emotional connection to a specific individual [1,2]. According to Bowlby’s attachment theory and Ainsworth’s research using the Strange Situation Procedure (SSP), attachment is conceptualized as a behavioral and representational system that organizes proximity seeking, secure-base use, and emotion regulation within the infant–caregiver relationship [1,2,3]. In particular, early attachment formation before the age of three is considered a sensitive period, significantly influencing not only the child’s social and emotional development but also all aspects of holistic development. During this period, the influence of the primary caregiver, usually the mother, is most pronounced [1,2].

One of the conditions that significantly hinders early attachment formation is autism spectrum disorder (ASD). ASD is a neurodevelopmental disorder characterized by qualitative deficits in language and communication, impaired social interaction, and restricted, repetitive behaviors [4]. ASD, which causes extensive social and emotional impairments, severely affects the ability to form relationships with others. Given these characteristics, early studies often concluded that children with autism could not form attachments [5,6] or were unable to establish secure attachments, and instead exhibited avoidant attachment and other pathological patterns [7]. However, beginning in the 1990s, when ASD came to be conceptualized not as a generalized social deficit but as a constellation of specific social impairments, many studies reported that children with ASD, even though the stability of attachment might be lower than in typically developing children, could form stable attachments if the mother was consistent and responsive to the child [8,9,10,11,12]. Therefore, the role of the primary caregiver, particularly the mother, is considered crucial in the attachment formation process for children with ASD.

However, the characteristics of children with ASD, such as qualitative impairments in interaction and communication, as well as hyper- or hypo-responsiveness to sensory stimuli, pose significant challenges [13,14,15] not only to the child’s attachment to the mother but also to the mother’s attachment to the child. These symptoms typically emerge during infancy and early childhood, the crucial period for initial attachment formation. During interactions with the primary caregiver, usually the mother, these children often exhibit difficulties such as lack of eye contact and failure to respond to their names. Additionally, mothers of children with ASD may struggle to accurately interpret their child’s messages or demands and may find it challenging to manage their child’s sensory responses [11,14,16,17]. When children show a lack of response or behaviors that differ from what the mother understands, mothers may feel rejected, which can hinder the development of a close emotional bond with their child [18,19,20].

In studies focusing on parents of children with ASD, many have highlighted the stress and challenges faced by the primary caregiver, usually the mother, with one recurring element being the difficulty in forming attachments with their child [21,22,23,24]. Additionally, psychological factors such as stress and depression, which are often experienced during the caregiving process, are found to negatively impact the mother’s attachment formation with the child, further hindering the development of a secure attachment [12,23]. The period when the symptoms of ASD begin to manifest coincides with the critical period for mother–child attachment formation, naturally leading to difficulties in forming attachments [25,26]. Therefore, given these circumstances, it can be inferred that the attachment experiences of mothers of children with ASD are significantly different from those of mothers with typically developing children.

To date, research on mothers’ attachment to children with ASD has primarily identified attachment-related variables and examined their associations with the level of maternal attachment. Findings indicate that maternal attachment varies with mothers’ perceptions and appraisals of the child’s condition [27], patterns of mother–child interaction [23,26], maternal sensitivity [10,23,28], parenting stress [11], depression [26], and mothers’ own attachment security in childhood [29].

These variations in the degree of attachment that mothers of children with ASD have towards their children appear to result from the attachment experiences that mothers undergo in their relationships with their children. These experiences can continually influence the mother’s caregiving practices [19]. Therefore, it can be assumed that the degree of a mother’s attachment to her child reflects the attachment process and experiences with the child.

However, research focusing on the attachment experiences of mothers of children with ASD and the process of forming attachment from the mothers’ perspective is scarce, leading to a lack of understanding of the attachment process of mothers towards their children with ASD. Furthermore, due to the limited understanding of the specific stages where mothers encounter difficulties in forming attachments, interventions aimed at enhancing maternal attachment are likely to face significant limitations.

Considering that the primary care for children with ASD is provided by their mothers, who play a pivotal role in the attachment development of their children, an in-depth and comprehensive exploration of the attachment processes of mothers caring for children with ASD is essential. Additionally, based on the results of previous studies, it is presumed that the attachment process with the child will vary depending on the degree of attachment the mother has towards her child with ASD. Therefore, research that examines the attachment process between mothers and their children according to the degree of attachment the mother has towards her child with ASD is expected to enhance the understanding of the attachment processes of these mothers.

Therefore, this study aims to investigate the degree of attachment of mothers of children with ASD and to examine the attachment process according to the degree of attachment through the application of grounded theory, a qualitative research method that focuses on understanding human experiences and interactions. This approach seeks to increase the specific understanding of the attachment process of mothers of children with ASD. Ultimately, it aims to provide an evidence-based foundation for developing systematic and individualized care plans and interventions that can assist mothers in forming attachments with their children.

## 2. Materials and Methods

### 2.1. Research Design

This study applies between-method triangulation by using both quantitative and qualitative research methods. Specifically, the research was conducted in two stages:

#### 2.1.1. Quantitative Phase

A cross-sectional survey was conducted to assess the degree of attachment between mothers and their children with ASD. This initial phase aimed to provide a broad understanding of the attachment levels in this population. Furthermore, this phase functioned as a preceding step toward the second-stage study examining the attachment process among mothers of children with ASD.

#### 2.1.2. Qualitative Phase

Following the survey, a qualitative study was implemented using the grounded theory methodology of Strauss and Corbin [30]. This second phase aimed to explore the attachment processes in depth, based on the degree of attachment identified in the first phase. Through this qualitative approach, the study sought to understand the nuanced experiences and processes of attachment formation between mothers and their children with ASD.

### 2.2. Participants

The participants of this study are mothers of preschool-aged children who have been diagnosed with ASD based on the DSM-5 criteria by a child psychiatrist. Additionally, the participants were selected based on their ability to read and communicate in Korean, have no cognitive impairments that would hinder comprehension of the study or participation, and provide informed consent. The first-phase recruitment was carried out in Korea through three child mental health clinics and child development treatment centers and one welfare center in Gyeonggi Province. These institutions are primary service hubs for preschool children with ASD, which facilitated efficient recruitment. The sample was non-probability, relying on clinic- and center-based convenience sampling with voluntary participation, and a total of 64 participants were enrolled.

The participants for the second phase of the study were selected from the first phase participants, specifically those who scored in the top 20% (227–245 points) and the bottom 20% (136–172 points) on the attachment scale with their children diagnosed with ASD. This constitutes purposeful extreme-case sampling [31], focusing on such polar types amplifies contrasts in the processes, contexts, and meanings associated with very high versus very low attachment scores, thereby increasing information power [32]. From this pool, only those who agreed to participate were recruited, resulting in the selection of six subjects in the top group and six subjects in the bottom group.

### 2.3. Research Instruments

#### 2.3.1. Maternal Attachment Measurement Tool

To measure the level of attachment in mothers of children with ASD, we used the Korean Maternal Attachment Inventory developed by Hwang [33]. This instrument is a self-report questionnaire comprising 50 items, categorized into eight factors: positive emotion (*n* = 11), seeking contact (*n* = 7), self-sacrificing generosity (*n* = 10), proximity-keeping behavior (*n* = 4), protection (*n* = 5), unity (*n* = 6), detachment (*n* = 4), and expectation (*n* = 3). Hwang’s validation study established content, construct, and criterion validity for this instrument and reported good reliability (split-half = 0.88; Cronbach’s α = 0.94; test–retest = 0.92) [33]. In this study, Cronbach’s α was 94. Permission to use the maternal attachment measurement tool was obtained from the original author via email. After a preliminary survey and review of the existing items, we sought the advice of one nursing professor and one psychiatrist to make necessary revisions and improvements. Experts validated the tool’s content, confirming all items’ relevance without modifications.

#### 2.3.2. The Childhood Autism Rating Scale (CARS)

To measure the degree of autism symptoms in children with ASD targeted in-depth interviews, the CARS, originally developed and revised by Schopler et al. [34] and translated by Kim and Park [35], was used. This tool is designed to assess the presence and severity of ASD. It consists of 15 items, with higher scores indicating a higher degree of autism severity. A score of 30 or above is the threshold for an autism diagnosis. According to Moon et al.’s meta-analysis, the instrument’s reliability was generally adequate, with internal consistency (Cronbach’s α) ranging from 0.82 to 0.95 and test–retest reliability from 0.91 to 0.99 [36]. In this study, Cronbach’s α was 0.95.

#### 2.3.3. General Characteristics

For the general characteristics of this study, a questionnaire developed by the researcher was used to understand the demographic characteristics of children with ASD and their mothers, as well as the characteristics related to the disease and treatment process of ASD. The questionnaire included items on the age, marital status, educational level, religion, employment status, number of children, pregnancy planning, age of the child with ASD, gender, birth order, and age at diagnosis of the child with ASD.

#### 2.3.4. The In-Depth Interview Questions

The specific research questions were constructed based on previous studies [8,9,10,11,12,13,14,15,16,17,18,19,20,21,22,23,24,25,26,27,28,29,33] to accurately reflect the attachment experiences and processes with the children. Initially drafted questions were modified and supplemented through preliminary interviews with three mothers of children with ASD. These revised questions were then reviewed by four nursing college professors and used as the tool for in-depth interviews. The research questions covered thoughts about the child, the degree and methods of expressing affection towards the child, interactions with the child, the mother’s reactions to the child, the mother’s circumstances during pregnancy and childbirth, the situations before and after the child’s ASD diagnosis, the mother’s relationship with her own parents during childhood, time spent with the child, the significance of the child to the mother, difficulties in the relationship with the child, and the future relationship with the child.

### 2.4. Data Collection Methods and Procedures

Data collection for this study was conducted after obtaining approval from the Institutional Review Board (IRB). The first stage of the study, which measured the degree of attachment, was conducted with participants recruited from three child psychiatry clinics and child development therapy centers located in Seoul and Gyeonggi-do, South Korea, as well as one welfare center. The participants were mothers of children diagnosed with ASD by a psychiatrist and who met the study criteria. Data collection took place in February 2017. The study was explained to the participants, and written consent was obtained. Participants were given a questionnaire on general characteristics and a tool to measure maternal attachment, which they completed and returned immediately.

For the second stage of the study, after collecting the first-stage questionnaires, maternal attachment was assessed using the Korean Maternal Attachment Inventory developed by Hwang [33], and the questionnaire took approximately 30–90 min to complete. Participants scoring 230 points or above and 170 points or below were invited for in-depth interviews. Six participants among the top 20% of participants, whose attachment scores ranged from 227 to 245, and six participants from the bottom 20%, whose attachment scores ranged from 136 to 172, were included in the study. To understand the attachment experiences with their children based on the degree of attachment, the researcher re-explained the purpose of the study to each group before proceeding.

Individual in-depth interviews and participant observations were conducted until theoretical saturation was confirmed, meaning no new categories were identified in the collected data. The time and place of the interviews were arranged to be comfortable and convenient for the participants, ensuring no disruptions. All interviews were audio-recorded with prior consent and transcribed by the researcher on the same day. Each participant was interviewed 1–3 times, with each session lasting between 50 min and 2 h and 30 min. Non-verbal messages such as facial expressions, gestures, attitudes, and the researcher’s impressions or the atmosphere were recorded in a memo notebook. The in-depth interviews were conducted using semi-structured questions prepared in advance based on previous research.

### 2.5. Data Analysis Methods

The data analysis for the first phase of the study was conducted using SPSS for Windows Version 28.0 (IBM Corp. Released 2021. IBM SPSS Statistics for Windows). Descriptive statistical methods were used to analyze the general characteristics of the subjects and their attachment levels to their children. To verify the attachment levels according to the general characteristics of the subjects, *t*-tests and ANOVA were utilized. For analyzing the attachment levels according to the degree of autism symptoms in the children of in-depth interview participants, *t*-tests were used. Additionally, Cronbach’s α coefficient was employed to verify the reliability of the measurement tools.

For the second phase of the study, data analysis aimed at understanding the experiences of attachment processes according to the attachment levels of mothers of children with ASD was conducted simultaneously with data collection. This analysis was performed using the procedures of open coding, axial coding, and selective coding as suggested by Strauss and Corbin [30]. These three coding methods are not separate and independent stages but are sequential, cumulative, and interactively related [30].

### 2.6. Ethical Considerations

This study was conducted after obtaining approval from the IRB of Korea University (Approval No.: 1040548-KU-IRB-15-261-A-1). The research proceeded following this approval. The participants were fully informed about the study’s purpose, the assurance of anonymity, the use of recordings and notes, the exclusive use of data for research purposes, the possibility of being selected for in-depth interviews in the future, and their right to withdraw from the study at any time if they did not wish to participate. After providing this information, written consent was obtained from those who agreed to participate in the study. Participants were given ample time to read the consent form thoroughly before signing, and two copies of the consent form were prepared so that both the researcher and the participant could retain a copy. Upon completion of data collection, a small token of appreciation was given to the participants, with different tokens provided for quantitative and qualitative studies.

## 3. Results

### 3.1. First Phase of the Study: Level of Attachment in Mothers of Children with ASD

#### 3.1.1. General Characteristics of Mothers and Children with ASD

The study participants consisted of 64 mothers of children diagnosed with ASD and 64 their ASD children. The general characteristics of these mothers and children with ASD are detailed in Table 1 below.

#### 3.1.2. Degree of Attachment in Mothers of Children with ASD

The degree of attachment in mothers of children with ASD is detailed in Table 2 below. The attachment scores for these mothers ranged from 136 to 245 within a possible score range of 50 to 250. The average score was 200.39, with a mean item rating of 3.92 (SD = 0.64), indicating that the attachment scores of mothers to their children with ASD were above the midpoint. Specifically, the mean item ratings for the eight subcategories of attachment are detailed in Table 2.

#### 3.1.3. Attachment Level According to General Characteristics of Mothers and Children with ASD

The degree of attachment according to the general characteristics of mothers and their children with ASD showed a statistically significant difference in the characteristic of planned pregnancy (*t* = 4.39, *p* < 0.001). Mothers who had a planned pregnancy (M = 209.71, SD = 20.34) had higher attachment scores compared to those who had an unplanned pregnancy (M = 183.78, SD = 26.34). However, no statistically significant differences were found in other variables, including the mother’s age, religion, education level, and employment status, or the child’s age, gender, birth order, and age at diagnosis (Table 1).

### 3.2. Second Phase of the Study: Level of Attachment in Mothers of Children with ASD

#### 3.2.1. General Characteristics of In-Depth Interview Participants and Their Children with ASD

The general characteristics of the mothers of children with ASD who participated in the in-depth interviews and their children are as follows (Table 3).

#### 3.2.2. Paradigm Model of Attachment in Mothers of Children with ASD

The analysis of attachment in mothers of children with ASD was conducted by categorizing them into high and low attachment score groups. The findings were reanalyzed using similar concepts and categories, and the results are presented using a paradigm model, as shown in Table 4. Throughout this process, 140 concepts were identified, which were then integrated into 38 subcategories and 17 higher-order categories.

**Figure 1:**
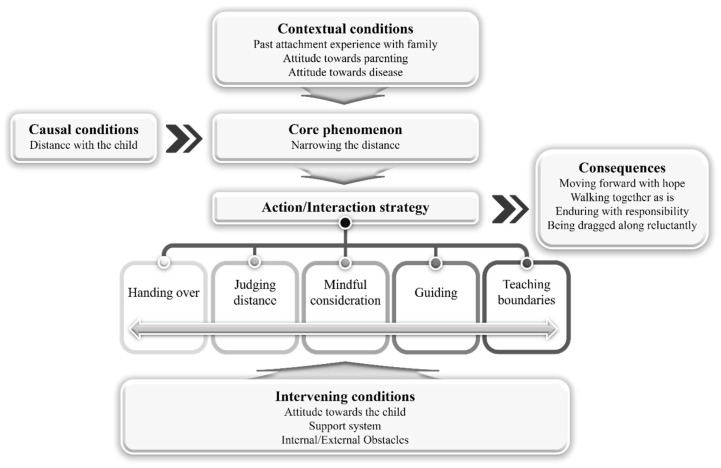
Paradigm Model of Attachment in Mothers of Children with ASD.

In the process of developing a grounded theory on the attachment process of mothers of children with ASD, the axial coding stage involves relating the categories that emerged during the open coding process. This involves connecting subcategories according to causal conditions, central phenomena, contextual conditions, intervening conditions, action/interaction strategies, and outcomes, as illustrated in Figure 1. For each of the identified categories, the range of properties and dimensions was presented.

##### Causal Conditions

Causal conditions refer to events or incidents that cause or influence a phenomenon. In this study, the causal condition identified was ‘distance with the child’. Participants perceived the differences in their children and prominently experienced decreased reciprocity, a symptom of ASD, in their interactions with their children. Despite this, participants felt a sense of responsibility and recognized their role as mothers of children with disabilities, forming the causal conditions for the attachment of mothers of children with ASD. The properties of ‘distance with the child’ were identified as <Presence> and <Degree>, with dimensional ranges of <Absent ↔ Present> and <Less ↔ More>, respectively.

“Even after a long time, eye contact continued to worsen, and there was hardly any improvement in speech. In fact, it got worse. Development was significantly delayed.” (Perception of difference—Slowness)—Participant 4

##### Core Phenomenon

The core phenomenon answers the question, “What is happening here?”, and refers to the central idea or event. In this study, the core phenomenon of the attachment process of mothers of children with ASD was identified as ‘narrowing the distance’. Participants had an initial distance with their disabled children but experienced special value in their children, such as loveliness, adorableness, pity, tenderness, and purity. They made efforts to emotionally and behaviorally get closer to their children, with thoughts and actions focused on supporting their children. The properties of ‘narrowing the distance’ were identified as <Degree>, with a dimensional range of <Less ↔ More>.

“I’ve always liked being busy, so I thought it would be difficult when I stopped working. At first, I did feel that way a bit, but there’s just so much to do in this area. There’s so much to find out, and I have to stay with my child and focus on him constantly. As a result, it fills me with a kind of passion.” (Willingness to bond—Immersion)—Participant 6.

##### Contextual Conditions

Contextual conditions influence the core phenomenon. In this study, the contextual conditions identified were ‘past attachment experiences with family’, ‘attitude towards parenting’, and ‘attitude towards disease’. The properties of past attachment experiences were identified as <Degree>, with a dimensional range of <Low ↔ High>. The properties of attitude towards parenting were identified as <Direction>, <Degree>, and <Amount>, with dimensional ranges of <Negative ↔ Positive>, <Low ↔ High>, and <Little ↔ Much>, respectively. The properties of attitude towards disease were identified as <Direction> and <Degree>, with dimensional ranges of <Negative ↔ Positive> and <Less ↔ More>, respectively.

“After OO was born, I went back to work. Since it was close to home and I only worked 3–4 days a week, I could still be involved in childcare. However, my mother-in-law did most of the childcare, and I took on a supportive role from behind, providing assistance and covering childcare expenses.” (Closeness in childcare—Auxiliary Role)—Participant 12.

##### Intervening Conditions

Intervening conditions are situational elements within the strategy structure that facilitate or inhibit the strategies. In this study, the intervening conditions identified were ‘attitude towards the child’, ‘support system’, and ‘internal and external obstacles’. The properties of attitude towards the child were identified as <Direction>, with a dimensional range of <Negative ↔ Positive>. The properties of the support system were identified as <Degree> and <Function>, with dimensional ranges of <Low ↔ High> and <Dissatisfied ↔ Satisfied>, respectively. The properties of internal and external obstacles were identified as <Degree>, <Presence>, and <Amount>, with dimensional ranges of <Less ↔ More>, <Absent ↔ Present>, and <Little ↔ Much>, respectively.

“My husband is so busy that I handle almost everything by myself, but my relationship with him isn’t bad. It’s just that I feel a bit lonely because he’s always so busy.” (Family conflicts—Unconsoled)—Participant 8.

##### Action/Interaction Strategies

Action/interaction strategies are designed to handle, regulate, and cope with the phenomenon observed in specific situations. In this study, the action/interaction strategies identified were ‘handing over’, ‘judging distance’, ‘mindful consideration’, ‘guiding’, and ‘teaching boundaries’. The properties of handing over, judging distance, mindful consideration, and teaching boundaries were identified as <Degree>, with dimensional ranges of <Less ↔ More>. The properties of guiding were identified as <Degree> and <Frequency>, with dimensional ranges of <Less ↔ More> and <Occasionally ↔ Frequently>, respectively.

“There are many things that OO can’t do skillfully. During those times, I encourage him by saying he can do it and patiently wait for him to do it slowly. I believe he can do it.” (Providing stability—Being Patient)—Participant 2.

##### Consequences

Consequences refer to the resolved states achieved through action/interaction strategies. In this study, the consequences identified were ‘moving forward with hope’, ‘walking together as is’, ‘enduring with responsibility’, and ‘being dragged along reluctantly’. The properties of moving forward with hope, walking together as is, enduring with responsibility, and being dragged along reluctantly were all identified as <Degree>, with dimensional ranges of <Less ↔ More>.

“I no longer keep in touch with my close high school friends. I just don’t want to hear them brag about their kids, and I don’t like talking about my own child either.” (Social isolation—Cutting Off Interactions with Others)—Participant 9

##### Core Category: Narrowing the Distance and Continuing the Connection

The core category of the attachment experiences of mothers of children with ASD was identified as ‘narrowing the distance and continuing the connection’. Perception of differences and decreased reciprocity are phenomena that manifest through the child’s disability. The child’s disability revolves continuously around the child, creating psychological and emotional distance between the mother and child. But the more positively the mother perceives the child’s disability, the larger the radius and the greater the number of holes in the sphere, allowing the mother to see the child more clearly. The mother’s efforts to narrow the distance with the child and to maintain a connection are key core categories that explain the attachment of mothers of children with ASD.

### 3.3. Attachment Process of Mothers of Children with ASD

Over time, the attachment process of mothers of children with ASD revealed overall stages such as the phase of confusion, exploration, close immersion, active engagement, perceptual exchange, stability, stagnation, and detachment. The attachment process differed between the high and low attachment score groups, and not all of the above-mentioned stages were present in each group; rather, only some of these stages were identified as part of the attachment process depending on the group.

#### 3.3.1. Stages in the Attachment Process of Mothers of Children with ASD

(1). ***Confusion phase*** begins when mothers perceive the differences in their children with ASD and confirm the diagnosis. As mothers increasingly realize that their children are different from others, they become overwhelmed with various emotions. They struggle to believe the situation, feel uncertain about what to do, and where to place their feelings, leading to growing despair.
(2). ***Exploration phase*** is when participants gauge the distance with their children and seek to get closer by examining and observing themselves, their children with ASD, and the surrounding environment. Through this observation, they broaden their interest in the child. In this phase, strategies primarily used include seeking information, attempting multi-faceted exploration, and employing internal regulation to measure the distance.
(3). ***Close immersion phase*** is when participants focus on narrowing the distance with their children by continuously checking, understanding, and reducing the child’s resistance. In this phase, participants predominantly experience positive emotions towards the child, and the increased interest in the child gained during the exploration phase further amplifies these positive feelings. The strategies primarily used in this phase include providing stability and empathy, encompassed within the broader strategy of mindful consideration.
(4). ***Active engagement phase*** is the most active and proactive stage in attempting to narrow the distance with children with ASD. During this phase, participants have the strongest will to promote the child’s growth using guiding strategies. Additionally, this phase is most influenced by the mother’s attitude towards the child. The more positively the mother perceives the child’s responses, the more active this phase becomes.
(5). ***Perceptual exchange phase*** is when mothers firmly perceive that some communication is occurring with their children with ASD. Mothers are confident in the responsiveness of the child to their expressions and actions. This phase is heavily influenced by the child’s functional level and improvement, as well as the mother’s sensitivity to the child. As mothers approach the stabilization phase, their positive feelings towards the child peak, leading to increased trust in the child.
(6). ***Stabilization phase*** is when participants establish a close and stable relationship with their children due to the narrowed distance. Participants feel comfortable in their relationship with the child and experience a sense of identification with the child, perceiving the child as a precious presence. In this phase, they observe the child’s growth and express hope for further improvement.
(7). ***Stagnation phase*** is characterized by settling into the current situation, employing strategies to teach boundaries. Participants do not harbor hopes or expectations for significant improvements in the child’s functional abilities. In this phase, participants express considerable concern and fear about the child’s uncertain future, which justifies the stagnation in their efforts to narrow the distance. This concern and fear act as a rationale for the stagnation in their efforts to further reduce the distance between themselves and their child.
(8). ***Detachment phase*** is characterized by a significant lack of attempts to narrow the distance with the child, with the distance gradually widening. Participants experience feelings of depression, distress, sadness, anger, and despair regarding their current situation and express anxiety and fear about the child’s future.

#### 3.3.2. Process in the High and Low Attachment Score Group

Participants in the high attachment score group, upon hearing the news of their child’s ASD, initially entered the confusion phase but relatively quickly moved on to the exploration phase. During the exploration phase, these participants naturally overlapped with the close immersion phase, working to enhance their understanding of themselves and their children. Subsequently, they reached the active engagement phase, where they continuously engaged in efforts to quickly narrow the distance with their children. However, some participants occasionally reverted to the exploration phase to further examine their children. After the active engagement phase, participants experienced the perceptual exchange phase, where they began to recognize some level of interaction with their children. While the duration of this phase varied among individuals, it ultimately led to the stabilization phase, where participants achieved a stable and secure attachment relationship with their children.

Participants in the low attachment score group also entered the confusion phase upon hearing the news of their child’s ASD. Although the duration of this phase varied among individuals, it was relatively long. They then moved on to the exploration phase. But some participants quickly reached the detachment phase instead, while others, with the intention of understanding their children, entered the close immersion phase. Subsequently, these participants reached the active engagement phase, driven by the hope for improvement in their child’s functioning and a strong sense of responsibility in their role as mothers. However, they generally did not stay long in this phase and soon reached the stagnation phase. Participants who quickly reached the detachment phase often experienced a cycle of returning to the confusion phase, then going through the exploration phase again, before eventually reaching the detachment phase once more.

#### 3.3.3. Attachment Types of Mothers of Children with ASD

In this study, the core category of ‘narrowing the distance’ in the attachment of mothers of children with ASD was identified in four types. Two types were found in the high attachment score group, and two types were identified in the low attachment score group (Table 5).

## 4. Discussion

This study, using a mixed-methods design, identified the attachment levels of mothers of preschool-aged children with ASD and the attachment processes associated with those levels. The grounded theory analysis yielded a core category—“maintaining connection while narrowing the distance”—and showed that the attachment process varied by level of attachment. We discuss these findings below.

### 4.1. Attachment Levels of Mothers with Children on the ASD

The study results showed that the mean score of maternal attachment was generally lower compared to the study by Hwang [33], who developed the attachment measurement tool used in this study. The mean score of attachment in Hwang’s study [33] was 219.5, higher than the mean score of 200.39 in the current study. This difference is likely because Hwang’s study targeted mothers of typically developing children, whereas this study focused on mothers of children with autism spectrum disorder. This suggests that characteristics of autism spectrum disorder in children significantly affect maternal attachment. And Bowlby [2] and Ainsworth et al. [1] viewed attachment as the bond from the child’s perspective, while some studies [37,38] emphasized that attachment should be seen from a bidirectional perspective. This study is significant as it assessed the attachment levels of mothers of children with autism from a bidirectional perspective. Notably, ‘Seeking Contact’ received the highest score, suggesting that mothers of children with autism prioritize contact with their children or consider it a relatively easier means of interaction, regardless of the severity of autism.

Additionally, the study found significant differences in attachment levels based on whether the pregnancy was planned, with unplanned pregnancies resulting in lower attachment scores [39,40]. This is notable as it highlights the relationship between pregnancy planning and maternal attachment specifically in mothers of children with autism. In this study, the K-CARS scores of autistic children ranged from 21 to 47, reflecting a wide range of functional levels within the autism spectrum [41]. There was no significant difference in maternal attachment based on the severity of the child’s autism. While reports suggest that higher levels of autism severity can lower the child’s attachment security [11,12,42] and reduce parent–child interactions [12,43], this does not necessarily explain the degree of maternal attachment. Therefore, the results of this study suggest that the functional level of autism does not have an absolute impact on maternal attachment [28,44]. In conclusion, this study is significant as it identifies factors influencing the attachment of mothers with children on ASD and discusses maternal attachment from the mother’s perspective, contrary to traditional literature focusing on the child’s bond.

### 4.2. Attachment Process and Types in Mothers of Preschool-Aged Children with ASD

This study employs a paradigm model to explain the attachment process of mothers with preschool-aged children diagnosed with ASD. The key category identified in the attachment process of these mothers is “narrowing the distance and continuing the connection.” Based on these findings, we discuss the characteristics of the attachment process and the types of narrowing the distance in mothers of children with ASD.

Firstly, the causal conditions affecting the attachment process of mothers with preschool-aged children with ASD are identified as the distance caused by the child’s ASD. Subcategories include “perception of difference,” “reduced reciprocity,” and “maternal awareness.” This aligns with previous studies [20,28,45] that have highlighted how the perception of difference and reduced reciprocity due to the child’s disability can influence the mother’s attachment formation. Several studies also emphasize that maternal awareness or identity is a fundamental factor in fostering emotional bonds with their children [45,46,47], confirming it as a causal condition in the attachment process. The central phenomenon in the attachment process of mothers with preschool-aged children with ASD, influenced by these causal conditions, is identified as “narrowing the distance” with their children. The presence of distance due to the child’s disability [13,14,15] leads mothers to engage in psychological and emotional efforts to reduce this distance and maintain their bond with the child. This is in contrast to the attachment process of mothers of typically developing children, which involves seeking proximity, affection, attunement, and commitment [18,33]. The presence of distance due to the child’s disability introduces a unique causal condition that differentiates the attachment experiences of mothers of children with ASD from those of mothers of typically developing children. This distinction underscores the unique challenges and experiences in the attachment processes of mothers of children with ASD, highlighting the need for tailored support and interventions to aid these mothers in fostering strong, positive attachments with their children.

The attachment process in mothers of children with ASD differs depending on the level of attachment. Participants with high attachment scores experienced a period of confusion regarding their child’s disability, but this period was relatively short. In contrast, participants with low attachment scores experienced a longer period of confusion. This variation is believed to be influenced by the maternal past attachment experiences, attitudes towards parenting, and perceptions of ASD, which were identified as intervening conditions in this study. Participants with high attachment scores generally had strong attachments to their own parents, high intimacy with siblings, and positive perceptions of their first encounter with their child. These mothers also exhibited high levels of parenting closeness, perceived fewer difficulties in parenting, and held positive views regarding their child’s illness and recovery. Conversely, participants with low attachment scores often lacked strong attachments to their parents, showed higher intimacy with siblings, or lacked such opportunities altogether. These mothers faced difficulties and stress during pregnancy and childbirth, leading to less positive initial encounters with their child. They also reported higher levels of parenting difficulties and often exhibited a more passive attitude towards their child’s illness, along with negative perceptions of ASD. From the perspective of ‘past attachment experiences with family,’ the attachment experiences with parents during childhood continue to influence the formation of attachment with their own children into adulthood. This aligns with the theory of intergenerational transmission of attachment [2]. Additionally, in terms of ‘parenting attitudes,’ it was reaffirmed that positive intentions and expectations towards pregnancy lead to positive attachment relationships with the child [40]. Parenting difficulties arising from maternal stress, anxiety, and sleep deprivation also affect attachment formation [12,48,49]. Moreover, it was confirmed that the degree of physical closeness in parenting influences the level of attachment a mother has towards her child, and a positive perception of the child’s illness without a passive attitude can facilitate attachment formation. These findings suggest additional factors to consider in future intervention studies aimed at strengthening maternal attachment.

Therefore, to enhance attachment in mothers of children with ASD, it is crucial to provide nursing services that help shorten the initial period of confusion. During the assessment process, it is important to closely examine past attachment experiences, parenting attitudes, and perceptions of the child’s illness. Positive aspects of past attachment experiences should be reinforced and transferred to the child. Counseling interventions should focus on the desired future attachment relationship with the child. For working mothers, it is essential to educate and raise awareness that while they may have less time for physical closeness, this does not equate to lower quality of attachment. Efforts should be made to connect them with all available resources to alleviate parenting difficulties.

Furthermore, the phases of exploration, close immersion, and activity may occur sequentially but can also happen simultaneously. It is crucial to recognize these phases as rapid progression may lead to burnout in the subjects. Particularly, in cases with low attachment scores, the transition from the exploration phase to the close immersion phase may not occur, leading instead to a phase of detachment. This indicates the necessity of coaching services for these subjects. Identifying significant internal and external obstacles and providing training programs to enhance the subjects’ resilience are essential interventions. Additionally, family interventions are necessary to minimize family conflicts, such as those with the husband or in-laws, and to ensure smooth functioning of the support system. It is also important to frequently check the health issues of the subjects to connect them promptly with medical and nursing services.

Next, this study classified the types of ‘narrowing the distance,’ the core phenomenon of the attachment process, into four categories. Among these, the high attachment score group included the active immersion type and the grateful contentment type, while the low attachment score group included the responsibility-centered type and the passive coping type.

Active immersion type is characterized by a high level of immersion in the ASD child, significantly narrowing the distance with the child. These mothers are hopeful about the child’s prognosis and generally exhibit positive attitudes towards their child’s growth and improvement. They actively use strategies such as understanding and guidingproactive attachment behavior aimed at alleviating the child’s symptoms. This strategy is often included in various attachment programs, involving activities like touching, engaging with the child, and increasing shared experiences, which can help in reducing symptoms and enhancing attachment in children with ASD [23,50]. However, providing education on the variability of symptoms with the child’s growth is necessary to prepare for future changes.

Grateful contentment type, similar to the active immersion type, focuses on satisfaction with the present rather than striving to enhance the child’s abilities. These mothers acknowledge that their child’s prognosis might not always be favorable and maintain an open mind. This is considered the most ideal attachment process type for mothers of children with ASD. They use a balanced mix of strategies such as gauging, understanding, and guiding. Understanding involves providing stability and empathy, linked to maternal sensitivity and empathy. Despite the communication difficulties associated with ASD, these mothers may demonstrate higher sensitivity, potentially leading to greater empathy. Previous interventions involving responsive listening, waiting, increased interaction, and empathic behavior have shown positive results in strengthening attachment [50,51].

Responsibility-centered type, based on strong maternal awareness and responsibility, focuses on the child’s upbringing but exhibits less intensity in narrowing the distance. The primary strategy used is boundary setting, which involves teaching the child about boundaries in social interactions. While this approach may align with behavior modification techniques for children with ASD [52], it is crucial to consider the emotional aspects of the mother during interventions.

Passive coping type shows the least degree of narrowing the distance, often using a strategy of resignation, leading to a passive acceptance of the current situation. Resignation involves observing or ignoring the challenging interactions with the child, reflecting the mother’s inner turmoil. This strategy can result in the child spending more time alone, which might exacerbate ASD symptoms [53]. This may particularly manifest in mothers who have low parenting satisfaction and are in a constrained environment that includes health issues, financial problems, and excessive burdens related to other family members [43,49]. Programs that promote the use of gauging, understanding, and guiding strategies should be provided to these mothers. These participants typically display negative attitudes towards their children, have low support systems, and face numerous internal and external obstacles. To enhance attachment, programs that foster positive perceptions of their children are necessary, beginning with a thorough understanding of ASD. Observing the child to identify key behaviors and expressions, as well as understanding the interaction between the child and their environment [12], can help mothers develop a better understanding of their children.

Furthermore, enhancing maternal attachment requires a robust social support system, including family, friends, and support networks [12,16], and reducing internal and external obstacles such as physical and mental health issues and economic problems [54]. Therefore, community-based programs that help establish effective support systems and minimize obstacles are needed. Specifically, the mother’s health issues pose significant barriers to building attachment with the child [54]. Hence, it is vital to include caregiver health management programs as part of family support programs for families with developmental disabilities.

## 5. Conclusions

This study aimed to develop a grounded theory that comprehensively explains the attachment process of mothers of preschool-aged children with ASD, who experience difficulties in attachment due to the characteristics of the disorder. Based on the results of this study, 64 participants were included in the quantitative analysis, and the top 20% and bottom 20% were classified according to their attachment scores. A qualitative study was then conducted to explore the attachment process of each group. As a result, it was confirmed that the attachment process of mothers of preschool-aged children with ASD is characterized by the core category of “maintaining connection while narrowing the distance.” In addition, the attachment processes differed between the high and low attachment groups, and overall, four distinct attachment types were identified.

Based on the findings of this study, identifying the needs of mothers of children with ASD while considering their attachment process and types, as well as developing and applying tailored intervention programs, could be useful in mediating attachment and related factors for these mothers. This study focused on mothers of preschool-aged children with ASD. However, because attachment is dynamic across the life course, there may be limitations in examining the attachment of mothers with ASD children of all ages.

Based on this study, we propose several recommendations. First, we recommend the development of tailored, attachment-informed interventions for mothers of children with ASD, grounded in the attachment processes and types identified in this study. Second, future research should examine maternal attachment across all developmental stages using qualitative and quantitative approaches. Finally, extensive policy research is needed to enhance the attachment of mothers of children with ASD through multifaceted approaches.

## Figures and Tables

**Table 1:** General Characteristics and Attachment Levels of Mothers and Children with ASD.

	Characteristics		N (%)	M ± SD	t or F	*p*-Value
Mothers (*n* = 64)	Planned Pregnancy	Yes	41 (64.1)	209.23 ± 19.88	4.39	<0.001
		No	23 (35.9)	183.78 ± 26.34		
	Age	30–34 years	17 (26.6)	196.71 ± 24.66	2.36	0.081
		35–39 years	34 (53.1)	196.41 ± 26.61		
		40–44 years	11 (17.2)	218.55 ± 20.09		
		≥45 years	2 (3.1)	199.50 ± 13.44		
	Marital Status	Married	63 (98.4)			
		Widowed	1 (1.6)			
	Religion	Christianity	30 (46.9)	200.70 ± 24.02	0.59	0.623
		Catholicism	15 (23.4)	204.40 ± 19.59		
		Buddhism	1 (1.6)	171.00		
		None	18 (28.1)	198.17 ± 32.92		
	Education Level	High school	5 (7.8)	209.60 ± 34.82	0.35	0.710
		College	46 (71.9)	199.78 ± 24.85		
		Graduate school	13 (20.3)	199.00 ± 26.80		
	Employment Status	Employed	28 (43.75)	194.50 ± 27.49	1.64	0.107
		Unemployed	36 (56.25)	204.97 ± 23.66		
	Number of Children	1	25 (39.1)			
		2	36 (56.2)			
		3	2 (3.1)			
		5	1 (1.6)			
Children (*n* = 64)	Age	3 years	4 (6.2)	208.75 ± 29.62	0.78	0.543
		4 years	14 (21.9)	198.79 ± 30.16		
		5 years	23 (35.9)	202.17 ± 21.67		
		6 years	11 (17.2)	189.45 ± 32.00		
		7 years	12 (18.8)	206.08 ± 20.45		
	Gender	Male	53 (82.8)	200.58 ± 26.10	0.13	0.896
		Female	11 (17.2)	199.45 ± 25.08		
	Birth Order	First	42 (65.6)	200.50 ± 23.49	0.019	0.981
		Second	20 (31.3)	199.85 ± 31.42		
		Third	2 (3.1)	203.50 ± 17.68		
	Age at Diagnosis (months)	≤24	5 (7.8)	211.20 ± 34.10	0.459	0.765
		24–36	29 (45.3)	196.41 ± 24.75		
		37–48	17 (26.6)	203.00 ± 27.31		
		49–60	9 (14.1)	203.33 ± 18.66		
		≥61	4 (6.2)	198.00 ± 36.19		

**Table 2:** Degree of Attachment in Mothers of Children with ASD.

Content	Number of Items	Mean	Mean Item Rating (SD)	Minimum	Maximum
Positive Emotion	11	46.91	4.26 (±0.44)	33	55
Seeking Contact	7	30.13	4.30 (±0.23)	20	35
Self-Sacrificing Generosity	10	36.25	3.63 (±0.64)	23	48
Proximity-Keeping Behavior	4	16.25	4.06 (±0.10)	9	20
Protection	5	20.50	4.10 (±0.20)	14	25
Unity (Cohesion)	6	24.97	4.16 (±0.33)	15	30
Detachment	4	10.17	2.54 (±0.40)	4	20
Expectation	3	10.64	3.55 (±0.76)	5	15
total	50	200.39	3.92 (±0.64)	136	245

**Table 3:** General Characteristics of In-depth Interview Participants.

Participant (*n* = 12)	Attachment Score	Age	Job	Education Level	Child’s Age	Child’s Gender	Birth Order	Diagnosis Age (Months)	Autism Severity	Interview Frequency
High Attachment Group										
1	241	40	None	High school	5	Male	1	27	33	3
2	230	31	None	High school	5	Male	2	45	24.5	1
3	237	34	Yes	Graduate school	4	Male	1	25	21	3
4	231	33	Yes	Graduate school	5	Female	1	38	32.5	2
5	245	42	None	College	4	Male	2	20	27.5	3
6	233	35	Yes	College	4	Male	1	36	22.5	2
Low Attachment Group										
7	154	37	Yes	College	6	Female	1	65	30	1
8	169	39	None	Graduate school	7	Female	2	39	26.5	2
9	136	38	Yes	College	4	Male	2	32	47	3
10	169	37	Yes	College	5	Male	1	36	44	1
11	168	34	None	College	5	Male	2	40	34	2
12	171	35	Yes	Graduate school	3	Male	1	31	21	2

**Table 4:** Conceptualization and Categorization of Data According to the Paradigm Model.

Concept	Subcategory	Category	Paradigm Condition
Uniqueness, Slowness, Suspicion, Oddness, Regression	Perception of difference	Distance with the child	Causal conditions
Feeling lost, Unresponsiveness, Frustration, Closed-off, Lack of communication	Decreased reciprocity		
Responsibility, Perception of motherly role	Maternal awareness		
Lovable, Cute, Pitiful, Special, Precious, Pure, Trust	Special value to me	Narrowing the distance	Core phenomenon
Contact, Continuation, Immersion, Helping thoughts	Willingness to bond		
Intimacy with the mother, Feelings of being loved, Sense of security, Resemblance, Reluctance to reverse, Feeling pushed away	Attachment perception with parents	Past attachment experience with family	Contextual conditions
Strong bond with siblings, Reliance on siblings	Intimacy with siblings		
Guilt, Anticipation, Suddenness, Bewilderment	Perception of first meeting with child	Attitude towards parenting	
Sole responsibility for childcare, Decreased shared time, Separation from child, Auxiliary role	Closeness in childcare		
Deviation, Strangeness, Sensitive child, Ease, Sleep deprivation, Extended work	Difficulty in parenting		
Trust in recoverability, Perception of incurable disease, Recognition of limits	Disease perception	Attitude towards disease	
Taking it lightly, Vague reassurance	Carefree attitude		
Natural reactions, Excessive contact, Child following, Increased child affection, Being used as a tool	Mother’s perception of child’s response	Attitude towards the child	Intervening conditions
Treatment avoiding permissiveness, Emotion-focused treatment	Core treatment direction		
Husband’s compensation, Shared childcare, Support from non-disabled siblings, Parental help, Sibling support	Family support	Support system	
Helping hands, Support from friends	Social support		
Husband’s criticism, Unconsoled, Indifference to child, Conflict with in-laws	Family conflicts	Internal/External obstacles	
Being sick, Caring for other children	Other care demands		
Burden of others’ views, Criticism from others towards child	Negative perception from others		
Unloving stereotypical expressions, Neglect, Muddle through	Passive handling	Handing over	Action/Interaction strategy
Seeking treatment information, Acquiring knowledge about illness, Sharing experiences	Information seeking	Judging distance	
Observing, Monitoring the child’s surroundings	Multi-faceted exploration		
Self-reflection, Recognizing my emotions	Internal regulation		
Being patient, Making the child comfortable	Providing stability	Mindful consideration	
Seeing as is, Reading emotions, Active listening	Empathy		
Kissing, Stroking, Rubbing, Hugging	Contact	Guiding	
Providing stimulation, Praising, Following professional advice, Recognizing signals, Responding immediately	Active involvement		
Spending time together, Engaging in community experiences	Being together		
Being firm, Not allowing, Limiting demands	Setting firm standards	Teaching boundaries	
One body with one mind, Valuing, Seeing as a gift, A source of strength, Joy, Pride, Contentment	Precious existence	Moving forward with hope	Consequences
Feeling like a real mother, Confidence, Changing perception of disability, Hope	Raising one’s head		
Increased expression of the child, Expanded expression of affection towards others, Emergence of new behaviors	Observing child’s growth		
Gratitude for the present, Satisfaction with the child	Living a content life	Walking together as is	
Comfort, Stability, Relief	Sense of well-being		
Destiny, Lifelong task, Acceptance	Seeing as fate	Enduring with responsibility	
Upset, Sadness, Anger, Anxiety, Despair, Uncertainty, Fear, Depression, Sense of disconnection	Emotional difficulties	Being dragged along reluctantly	
Pain all over, Sleeplessness, Feeling physically drained	Physical exhaustion		
Withdrawal, Cutting off interactions with others, Feeling comfortable in unfamiliar places, Hiding child’s disability, Reluctance to go out	Social isolation		

**Table 5:** Comparison of ‘Narrowing the Distance’ by Attachment Type.

Type	High Attachment Score Group		Low Attachment Score Group	
	Active Immersion	Grateful Contentment	Responsibility— Centered	Passive Coping
Participant	1, 4, 6	2, 3, 5	7, 8, 10, 12	9, 11
Causal Conditions				
Distance from Child	Exists	Exists	Exists	Exists
Perception of Difference	Exists	Exists	Exists	Exists
Reduced Reciprocity	More → Less	More → Less	More → Less	More
Maternal Awareness	More	More	More	Moderate
Core Phenomenon				
Narrowing the Distance	More	More	Less	Less
Contextual Conditions				
Past Attachment Experiences with Family				
Attachment Experience with Own Parents	High	High	Moderate to Low	Low
Attitude towards Parenting				
Perception of First Meeting with Child	Positive	Positive	Positive to Negative	Negative
Degree of Parenting Closeness	High	High	Moderate	Moderate to Low
Parenting Difficulties	More → Less	More → Less	More → Less	More
Attitude towards Illness	Positive	Positive to Moderate	Positive to Negative	Negative
Intervening Conditions				
Attitude towards Child	Positive	Positive	Moderate to Negative	Negative
Support System	High	High	High to Low	Low
Internal and External Obstacles	Few	Few	Many to Few	Many
Action/interaction strategies	Judging distance (Moderate to More) Mindful consideration (More) Guiding (More)	Judging distance (More) Mindful consideration (More) Guiding (More)	Guiding (Moderate) Teaching boundaries (More)	Handing over (More)
Consequences	moving forward with hope	walking together as is	enduring with responsibility	being dragged along reluctantly

## Data Availability

The original contributions presented in the study are included in the article, further inquiries can be directed to the corresponding author.
